# Prêt-à-Jouer: commercial videogames as ready-made human perception labs

**DOI:** 10.3389/fcogn.2026.1944577

**Published:** 2026-09-03

**Authors:** Jaap Munneke, Brian Anthony, Jennifer E. Corbett

**Affiliations:** 1 Institute for Cognitive and Brain Health, Northeastern University, Boston, MA, United States; 2 Institute for Medical Engineering and Science, Massachusetts Institute of Technology, Cambridge, MA, United States; 3 Computer Science and Artificial Intelligence Lab, Massachusetts Institute of Technology, Cambridge, MA, United States

**Keywords:** behavioral science, cognition, commercial video game, ecological validity, eye tracking, perception, repeated measures designs, research methods

## Abstract

Cognitive science has long relied on carefully controlled laboratory paradigms to study perception, attention, and executive functioning, raising persistent questions about ecological validity when scenes are complex, demands are self-paced, and outcomes matter to the observer. Commercial videogames present exactly this kind of environment. They are cognitively demanding by design, motivating by nature and consistent enough across players to support systematic behavioral comparison. Unlike the tools of typical laboratory research, they already exist. Here, we encourage researchers to treat commercial games as ready-made testing grounds requiring only a minimal experimental toolkit of screen recording, eye tracking, and behavioral timing. We describe how researchers can select games, generate testable perceptual hypotheses directly from a game's affordances, and implement within-subjects designs, with no engine access or developer cooperation required. We close by distinguishing the ecological questions this approach can answer from the mechanistic ones it cannot.

## 1 Introduction

Every year, thousands of studies investigate perception, attention, and executive functioning using tasks that reduce the visual world to its essential variables. Participants respond to simplified stimuli stripped of real-world complexity, such as in a flanker (Eriksen and Eriksen, 1974) or a spatial cueing paradigm (Posner et al., 1980). This approach follows a clear logic. Reducing the world to a small number of controlled variables makes it possible to isolate perceptual mechanisms with precision that natural scenes cannot offer. It also raises the old question of whether laboratory psychology can produce findings that speak to perception as it operates outside the laboratory (Neisser, 1976). This problem is not simply philosophical. Converging evidence shows performance on certain cognitive tasks fails to predict real-world functioning that relies on the studied cognitive mechanism (Burgess et al., 2006; Chaytor et al., 2006; Chaytor and Schmitter-Edgecombe, 2003; Rosenholtz, 2025). Commercial videogames represent promising solutions for expanding our experimental toolkit vs. replacing what already works.

On any given day, millions of hours of gameplay are logged across the world (Bavelier et al., 2010; Przybylski and Weinstein, 2019). Players navigate 3D environments, track multiple moving objects, switch rapidly between tasks, form spatial representations of virtual worlds, and make decisions with significant in-game consequences. Crucially, each of these demands is a direct reflection of deliberate game design choices. A commercial game contains a controlled, repeatable stimulus stream and has an engaged, motivated observer. However, it does not have an event log. This is where a set of tools developed for research on how people use interactive systems becomes particularly useful. As we will describe, screen recording, eye tracking, and configuration of a game's built-in options can be applied to any commercial title exactly as it ships, with no access to source code.

We do not advocate abandoning laboratory paradigms, which remain indispensable for mechanistic inquiry. Instead, we encourage researchers to complement them using commercial video games as inherently motivating, sustained environments where genuine cognitive demand arises directly from gameplay structure. We first describe how games can enhance ecological validity in the lab. We then review what studies using commercial games have already revealed about perception and cognition. Finally, we outline an experimental toolkit for converting a game's design into variables that test perceptual and cognitive hypotheses.

## 2 Ecological validity

Most commercial games are designed to be engaging, which means that they impose sustained, varied, and often intense cognitive demands on the player. A player navigating a fast-paced 1st-person shooter must continuously parse a cluttered visual scene, suppress irrelevant distractors and track multiple moving targets. A player managing a large military in a real-time strategy game must maintain and update multiple representations in working memory, plan several moves ahead, and switch flexibly between task demands. These are not approximations of cognitive constructs, but natural instantiations, embedded in a context that is richer and more dynamic than any laboratory task could reasonably replicate (Green and Bavelier, 2012).

Commercial games also provide an ecologically grounded source of individual differences in expertise, strategy, and cognitive style, expressed continuously throughout gameplay rather than in isolated probe responses. Along these lines, a common objection to naturalistic research is that unconstrained environments vary too much to allow systematic comparison (McMahan et al., 2011). Games are far richer environments than laboratory displays, but they are not necessarily less consistent. They are standardized environments in that a given level or map typically presents the same stimuli, the same visual clutter, and the same spatial layout, allowing for a level of reproducibility that true naturalistic research often struggles to attain.

The ecological validity and reproducibility of commercial games stem directly from the *affordances* or action possibilities games offer users (Gibson, 1979; Norman, 2013). This affordance structure makes game environments theoretically tractable as research environments. Elements within them draw the player's eye to particular locations, reward specific response patterns, and impose sequences of decisions. These affordances emerge from deliberate design choices about what the game demands of its player (Corbett and Munneke, 2025). Crucially, these demands can be mapped onto cognitive constructs without requiring access to the game's underlying code. A researcher does not need to know how the game engine calculates enemy behavior to observe that a player of a fast-paced action game must rapidly detect and respond to threats appearing in the visual periphery. This is a naturalistic example of stimulus-driven exogenous attentional behavior (Posner et al., 1980), where the affordance structure of the game does the work that an experimental manipulation would otherwise require of the researcher.

## 3 Previous work

Games have already provided a productive environment for studying perceptual learning, attention, and executive functioning. Foundational work by Green and Bavelier (2003) demonstrated that habitual players of action video games outperformed non-players across multiple attentional paradigms, including the attentional blink, multiple object tracking, and the spatial distribution of attention. Non-players trained on a fast-paced 1st-person shooter showed marked improvements on these tasks, while a group trained on a visually simple puzzle game did not (Green and Bavelier, 2012). These findings are contrary to the specificity typically observed in laboratory perceptual learning tasks, suggesting that the affordance structure of the action game was the driving factor. Subsequent work extended these findings to lower-level perception including visual acuity, contrast sensitivity (Green et al., 2010), and aspects of spatial cognition like mental rotation (Spence and Feng, 2010). Successive meta-analyses confirmed that perception and top-down attention are among the cognitive domains most reliably enhanced by action game experience, with effect sizes among the largest in the cognitive training literature (Bediou et al., 2018, 2023).

Executive functioning shows a similar but more component-specific pattern. Working memory, cognitive flexibility, and inhibitory control (Miyake et al., 2000) each respond differently to different games. Training on real-time strategy games has produced improvements in task switching and working memory in older adults (Basak et al., 2008) and selectively enhanced cognitive flexibility (Glass et al., 2013), while training on spatial and shooter titles has produced corresponding gains in spatial and visual working memory (Blacker et al., 2014; Oei and Patterson, 2013). A meta-analysis confirmed that specific design features vs. genre labels are better predictors of which executive functions a given game will train (Dale and Shawn Green, 2017), and inhibitory control shows a detectable relationship to in-game behavioral metrics like actions per minute (Jakubowska et al., 2021).

Although commercial games are already being used as tools to test cognitive and perceptual hypotheses, we lack a systematic account of how to extract and manipulate specific perceptual and cognitive signals as dependent and independent variables over the course of gameplay.

## 4 Experimental toolkit

Studying perception inside a commercial videogame presents an immediate practical challenge. Researchers have no access to the game's internal architecture (Sobczyk et al., 2015). There are no event files, no millisecond-precise stimulus logs, no parameter controls. However, the researcher does have everything visible and measurable from the outside including the screen and the player, as well as the ability to select particular games that target cognitive and perceptual constructs of interest and manipulate the built-in options that players can use to customize gameplay.

*Screen* recording is an accessible yet underutilized tool in this context. In Human-Computer Interaction (HCI) research, screen recording and interaction logging routinely evaluate interface navigation and performance breakdowns (Carroll, 1997). In the context of cognitive science, the same data stream can be used to index a range of constructs such as inhibitory control through failures to suppress a prepotent response (Verbruggen and Logan, 2009), cognitive flexibility through shifts in strategy following negative feedback (Monchi et al., 2001), and prospective memory through a player's return to a location or objective after an interruption (Einstein and McDaniel, 1990).

As illustrated in Table 1, a continuous recording of the game display captures the full behavioral output of the player over time, including every decision, every movement, and every error. Many gameplay events offer natural choice-reaction opportunities, such as sudden threats or navigation forks. Response latencies extracted through frame-by-frame analysis provide ecologically valid reaction times without game modification or player awareness. Additionally, the outcomes of actions can often be scored directly from recordings, such as whether a player successfully responds to a threat, chooses the correct path, or misses a target entirely. With careful *post-hoc* event coding, screen recordings yield a rich reconstruction of dependent variables used in perception and cognition research like decision timing, response latencies, error patterns, strategy switches, and spatial navigation choices shaped by the game's affordance structure. Corresponding independent variables can be extracted by mapping built-in game features. Decision timing varies as a function of the number of choices or items, response latencies are affected by the perceptual and attentional demands surrounding the triggering event, error patterns change as a function of difficulty, players switch strategies in response to observable changes unfolding over the course of gameplay, and their spatial navigation choices vary as functions of the complexity and familiarity of the environment.

**Table 1:** Examples of dependent variables measurable from screen recordings and their corresponding built-in independent variables (game affordances).

DVs	DV examples	IVs	IV examples
Decision timing	Time to choose a route; time to select a weapon; time to spot and collect a resource; time to choose to engage or retreat	Number of choices or items available	Number of viable routes at a fork; number of enemies or visible threats; presence or absence of new information; visibility conditions at the decision point; resource scarcity at the moment of choice (e.g., reloading)
Response latency	Braking time to an obstacle or corner; time between an enemy appearing and the player firing or taking cover; time between a health warning/indicator and the player's response; time to dodge or parry an incoming attack	Distance, difficulty, and salience of the triggering event	Distance or warning time before an obstacle or enemy appears; difficulty of the level (enemy reaction speed, aggression); level segment type (open ground vs. chokepoint); concurrent task load (e.g., driving while monitoring a course map); salience of the triggering cue (flashing indicator vs. peripheral, subtle cue)
Error pattern	Crashes, spinouts, or missed braking points; missed jumps or falls; friendly fire accidents or misidentified targets; running out of ammo or resources; wrong turns or dead-end backtracking during navigation	Level of difficulty	Enemy density; environmental conditions (e.g., night visibility); difficulty settings; simultaneous or multitasking demands
Strategy switch	Shifting from attack to defense after an event; abandoning a plan after a scouting report; changing target priority mid-task in response to a new threat; switching from a stealth to a direct engagement approach	Observable changes unfolding over the course of gameplay	Opponent behavior changing from passive to aggressive; negative feedback events (e.g., near crashes); level or phase transitions (e.g., a boss with different attack patterns); resource depletion (e.g., running low on fuel)
Spatial navigation choice	Wide entry vs. apex-hugging when rounding a corner; shortcutting vs. safe route selection; cover-to-cover movement vs. open ground crossings; exploration and backtracking frequency	Complexity and familiarity of the environment	Level layout (number of alternative paths); terrain type (open ground vs. dense cover); previously explored vs. novel areas; visibility conditions along potential routes

*Eye tracking* captures a rich behavioral stream from gaze rather than action, with standard metrics extracted automatically via software rather than manual coding. In HCI, eye tracking routinely evaluates interface design, tracking whether players notice critical information or follow intended attentional guidance (Montolio-Vila et al., 2024). Eye-tracking metrics can also index a range of perceptual and cognitive constructs using commercial video games. For example, selective attention and processing load can vary as functions of the salience and placement of on-screen elements as reflected by changes in fixation location and duration (Meghanathan et al., 2015). Oculomotor efficiency and arousal can be indexed by saccadic velocity (Di Stasi et al., 2013), the breadth of attention across the visual field can be inferred from gaze distribution (Jeong et al., 2022, 2024), and both can be modulated by player expertise and scene complexity. Visual search efficiency in terms of the number of saccades required to locate a target varies as a function of the density and clutter of the visual scene (Phillips and Edelman, 2008). Attentional engagement can be measured with saccadic reaction time and affected by the sudden appearance or salience of a triggering event (Kingstone and Klein, 1993). Cognitive load derived from pupil diameter varies with difficulty tier and the number of simultaneous demands imposed on the player (Beatty, 1982; Kahneman and Beatty, 1966). Scene parsing and the balance of top-down vs. bottom-up control can be indexed by scan path shape and change depending on whether an active goal or a salient but irrelevant event is present (Henderson, 2003).

*Game selection & configurable options:* Screen and eye recordings afford a wide range of typical perceptual and cognitive variables inherently available commercial games. However, the measures discussed so far index quasi-independent variables that occur naturally within a game rather than variation researchers deliberately manipulate. These passively obtained measures do not by themselves guide a researcher in selecting a game before data collection, nor do they indicate which configurable options within that game could be directly manipulated to test specific hypotheses. At present, researchers largely select games intuitively based on apparent relevance or precedent, without explicit structural guidance. However, design features predict cognitive outcomes far better than broad genre labels alone (Dale and Shawn Green, 2017). For example, two games classified as action titles can place entirely different demands on perception. In addition to categorical guidance by genre, we need more direct mapping from a game's affordances onto perceptual and cognitive constructs to guide researchers in selecting specific games because they structurally recruit the process under investigation. Because a player's previous experience with a given game is also.

Most commercial games ship with settings that function as ready-made independent variable manipulations requiring no code access or developer cooperation to implement. When narrowing options based on a game's general affordances, it is especially important to identify relevant configurable settings already built into that title. As illustrated in Table 2, constructs like spatial navigation strategy might guide a researcher toward open-world or navigation-heavy titles. Titles can then be specifically evaluated for relevant configurable options like mini-map presence and zoom level, and/or camera perspective toggle. These settings then become specific levers for manipulating a player's reliance on allocentric map-based vs. egocentric 1st-person representations of the environment. For example, as allocentric reliance decreases, route selection should become less direct, backtracking frequency should increase, and gaze should more often shift from the map toward peripheral landmarks, all trackable using screen recording and eye tracking.

**Table 2:** Examples mapping perceptual and cognitive constructs to game selection affordance criteria and corresponding built-in configuration options.

Construct	Game selection for affordance	Configurable options	What the options manipulate
Figure-ground segmentation, visual search	Games with camouflaged or low-contrast targets (e.g., stealth games, military shooters with concealment mechanics)	Colorblind or high-contrast accessibility mode, enemy/target outline highlighting toggle	Visual salience of the target against the background
Perceptual load	Games with tiered difficulty systems (e.g., action-adventure or roguelike games)	Initial difficulty level setting (e.g., novice, intermediate, advanced)	Enemy density, reaction speed, environmental visibility
Response-effect compatibility	3rd-person games with configurable control mapping	x- and/or y-axis inversion	Mapping between controller input and on-screen movement direction
Spatial distribution of attention	Games with wide, information-rich fields of view (e.g., open-world action games, flight or racing simulators)	Field of view setting (most reliably adjustable on PC, less consistently exposed on console)	Amount of peripheral visual information available at any given moment
Working memory, cognitive load	Games requiring players to track objectives, resources, or status information (e.g., real-time strategy or survival/resource-management games)	Heads-up display toggle	Whether information is displayed externally or must be held in memory
Spatial navigation strategy	Open-world or navigation-heavy games	Mini-map presence, zoom, camera perspective toggle	Reliance on allocentric (map-based) vs. egocentric (1st-person) spatial representations
Visuomotor control, isolated from perceptual detection	Precision-aiming games (e.g., 1st-person shooters)	Aim assist	Precision demands placed on visuomotor control

Our previous work (Corbett and Munneke, 2025) offers an illustration of this quasi-experimental logic. We examined players' existing preference for inverting the y-axis on their gaming controllers, the setting that controls the vertical orientation of the camera relative to the avatar. Y-axis inversion mainly concerns how users employ the right joystick for rotational movements like surveying the environment and focusing on target locations. Although some also invert the y-axis on the right joystick in 1st-person games where the user views only the environment as the avatar, users typically retain the default, non-inverted configuration. We focused specifically on 3rd-person games because y-axis inversion primarily involves response-effect compatibility between the motion of the joystick (e.g., “forward” or “up”) and the corresponding directional perception of the avatar's view (e.g., “look up”). We found this response-effect preference was linked to individual differences in how quickly people could mentally rotate novel shapes and how well they could overcome stimulus-response compatibility effects when the side of the response on the controller was opposite to the side of a target on the screen (the Simon Effect; Simon and Wolf, 1963).

These initial quasi-experimental results establish that the built-in configurable option to invert the right joystick's y-axis in commercial games can carry meaningful individual difference information even when treated as a self-selected preference. Future work can now build from these findings by directly manipulating y-axis inversion settings to measure the effects of response-effect compatibility on a range of cognitive and perceptual constructs related to mental rotation speed and stimulus-response compatibility, such as camera-orienting response latency, error rates when aiming or navigating under inverted vs. non-inverted mapping, pupil dilation during the adaptation period when switching between mappings, and the rate at which performance recovers as players adjust to an unfamiliar control scheme. This is one of many examples of how our toolkit can guide a researcher to select which affordance in a given game and which configurable option within that game are most expected to manipulate the perceptual or cognitive process of interest, then test that prediction with screen recording and eye tracking measures.

Crucially, because these configurable settings can be toggled instantly within the same session, this toolkit enables researchers to transition from traditional between-subjects training designs to high-powered, within-subjects repeated measures. Researchers can directly track how an individual's gaze distribution, response latency, or error patterns shift in real time as environmental affordances are actively manipulated.

## 5 Discussion

This perspective encourages researchers to treat commercial videogames as ready-made laboratories for perception and cognition research. The affordance structure in commercial games imposes genuine, sustained perceptual demands without any need for the researcher to orchestrate ecologically valid experimental conditions. A growing body of work has already taken advantage of commercial video games to examine how gameplay effects a variety of perceptual and cognitive factors. Here, we offer a toolkit for using screen recording, eye tracking, and configuration of a game's built-in options, as concrete, practical methods for turning commercial titles into sources of testable hypotheses.

This toolkit measures and manipulates standard laboratory variables using built-in design features rather than experimenter-engineered code. Beyond convenience, this approach offers several practical advantages. First, because players experience manipulations as ordinary parts of gameplay rather than as instructed experimental probes, the resulting measures avoid demand characteristics that can accompany explicit timing instructions or known test conditions in laboratory settings (Iarygina et al., 2025). Furthermore, because engagement with a commercial game is intrinsically motivating, our toolkit sidesteps the recruitment and compliance problems that otherwise limit sustained laboratory testing, particularly for effortful or lengthy protocols. Finally, by shifting from traditional between-subjects training designs to within-subjects repeated measures, this approach drastically improves statistical power while providing an elegant baseline control for individual differences.

This approach has clear limitations. Without access to a game's engine, some questions remain out of reach. The exact millisecond timing of a player's response, the precise parameters behind a perceptual manipulation, and the internal state of the game at any given moment may not be directly observable. Even when a configurable option like difficulty tier is manipulated deliberately, its underlying implementation remains opaque and coarser than a classic laboratory experimenter-defined parameter. These constraints mean our toolkit is best suited for ecological questions like how perception operates in a rich and self-motivated natural setting vs. mechanistic questions about precise computations underlying a perceptual operation. Laboratory paradigms remain the better tool for the latter. The two approaches are best understood as complementary, with commercial games supplementing traditional methods by testing whether effects established under controlled conditions generalize to richer, self-motivated settings.

Realizing the value of this approach at scale requires some shared practices across the field. Pre-registering the game and affordance selected, configurable options manipulated, and resulting hypotheses and analysis plan can help to shape resultant effects as cumulative rather than anecdotal. Standard eye-tracking protocols and calibration, agreed conventions for coding screen recordings, and open sharing of recorded sessions would all help as well. As in the studies discussed here, researchers should adopt the customary practice of measuring participants' prior experience with each specific game's UX, allowing them to control for differences through screening, counterbalance it across conditions, or otherwise include it as a covariate. None of this requires new infrastructure - only applying standards that perception and cognition research already uses.

Commercial videogames are among the richest sources of naturalistic visual behavior that we can use in the lab. People choose to spend time in these environments, which removes the motivational problem that has always complicated laboratory research. Perception scientists have historically treated this behavior as something to be gamified, replicated, or set aside as too uncontrolled to study. Our perspective is simpler. Commercial games do not require redesigning, licensing, or reverse engineering to answer perceptual questions. They require only a screen recorder, an eye tracker, and a researcher willing to state, in advance, which affordance and which configurable options are expected to reveal which perceptual processes.

## Data Availability

The original contributions presented in the study are included in the article/supplementary material, further inquiries can be directed to the corresponding author.
